# Competitive Designs at Burnley

**Published:** 1914-06-27

**Authors:** H. Percy Adams

**Affiliations:** 28 Woburn Place, Russell Square, London, W.C.


*To the Editor of* The Hospital.

### Competitive Designs at Burnley.

*To the Editor of* The Hospital.

Sir,—My attention has just been called to a notice on pp. 286-7 of The Hospital with reference to a competition being held for the proposed extension of the Burnley Victoria Hospital. I do not know where your correspondent obtained his information from, but the account is most inaccurate and misleading. As a matter of fact the committee of the hospital came to London and after exhaustive inquiries appointed me as assessor for this competition (by the way, my name is not Burton, but Percy). The conditions and specifications were most carefully drawn up by the committee and myself after my visit to the hospital, and I do not think they contain any fancies of individual members of the honorary staff. The specifications, which you have evidently never seen, contain full details of what is required and the amount of money available, and the decision as to the best design submitted is entirely in my hands. The committee have, in my opinion, done everything possible to obtain the best architect to carry out their hospital extensions, and your notice is (and I feel sure you will agree when you know the facts) most unjust and grossly misleading.

Faithfully yours,

H. Percy Adams.

28 Woburn Place, Russell Square, London, W.C.,
June 23, 1914.

[The printer's error in giving Mr. Adams a Christian name other than that which all the world knows we much regret. As to the rest of the paragraph, if Mr. Adams will kindly refer to it again he will see that the statement of the names of the architects at Burnley is followed by a general criticism of hospital competitions, as such recurring phrases as "the past has shown," "hitherto few hospitals," and "the tendency has been" abundantly show. The question with which the paragraph concluded he answers only by implication.—Ed. The Hospital.]

[Owing to pressure on our space a long letter on "Institutional Defalcations" has had to be held over till next week.]

